# Metastatic Syringocystadenocarcinoma Papilliferum: A Case Report, Tumor Genomic Profiling, and Literature Review

**DOI:** 10.1155/2020/9056209

**Published:** 2020-08-08

**Authors:** Erdem Altunel, Aleksandr Perepletchikov, Olga Kozyreva

**Affiliations:** ^1^Department of Medicine, Saint Elizabeth's Medical Center, Boston, Massachusetts, USA; ^2^Department of Medicine, Division of Medical Oncology, Dana Farber Cancer Institute at Saint Elizabeth's Medical Center, Boston, Massachusetts, USA; ^3^Department of Pathology, Saint Elizabeth's Medical Center, Boston, Massachusetts, USA; ^4^Department of Pathology, Tufts University School of Medicine, Boston, Massachusetts, USA

## Abstract

Syringocystadenocarcinoma papilliferum (SCACP) is an extremely rare cutaneous neoplasm of the apocrine or eccrine sweat glands. Solid and cystic glandular structures with cribriform and tubular architecture along with CK5/6, pankeratin and p63 immuno-profile set apart SCACP from other cutaneous malignancies. Wide local excision (WLE) has been the mainstay treatment for localized SCACP; however, no standard treatment has yet been established for unresectable or metastatic disease. Herein, we report a 74-year-old male with SCACP, who initially presented with a painful nodule on the upper back and later developed metastatic disease. He was treated with carboplatin and paclitaxel with concurrent intensity-modulated radiation therapy (IMRT), which resulted in disease stabilization for 12 months. Next generation sequencing (NGS) revealed a total of 18 genomic alterations associated with potential benefit from targeted therapeutics. PD-L1 expression was identified in 70% of tumor cells. These findings suggest that the opportunity of targeted therapeutics and immunotherapy exist as for metastatic SCACP. Reporting molecular profile of the rare tumors with no established standard treatment options should be encouraged.

## 1. Introduction

SCACP is an extremely rare cutaneous adnexal neoplasm, of which only less than 50 cases have been described in the literature since its first description in 1980 by Dissanayake et al. [1]. SCACP is originated from apocrine or eccrine sweat glands and often presents as a nodular mass in the scalp as well as other parts of the body [2]. Although its histopathologic features have been well described [3–5], the optimal treatment has not yet been established because of the rarity of the tumor. WLE has been the preferred treatment for localized SCACP [6]; however, there is no standard systemic therapy for the unresectable or metastatic disease. In addition, the genomic profile of SCACP has not yet been reported. Herein, we report a case of metastatic SCACP, its genomic profile, PD-L1 status, and response to the multimodal treatment.

## 2. Case Presentation

**Figure 1:**
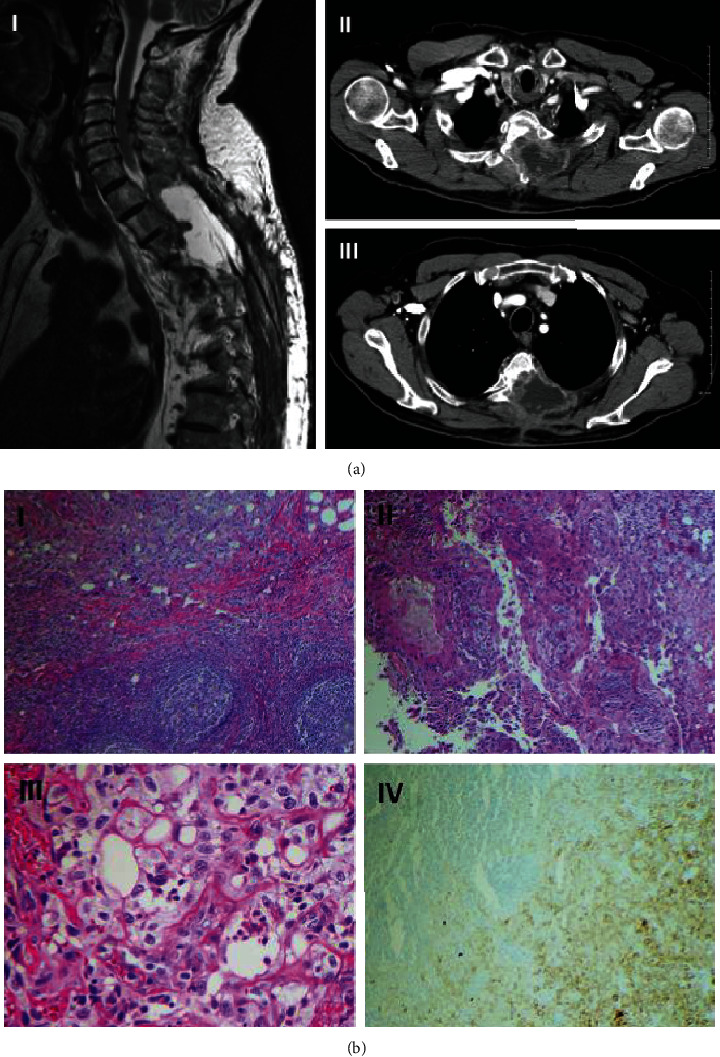
Radiologic and histologic appearance of syringocystadenocarcinoma papilliferum. (a) (I) Magnetic resonance imaging showing soft tissue mass in the thoracic spine. (II) Axial computed tomography (CT) scan showing tumor invasion into the vertebrae at T4 level. (III) Axial CT scan showing tumor invasion into the vertebrae at T2 level and enlarged right axillary lymph node. (b) (I) Lymph node with metastatic tumor, H&E stain, 40x. (II) Solid and glandular structures with warty architecture, lined by poorly-differentiated epithelium, H&E stain, 100x. (III) Clusters and nests of highly pleomorphic cells with bizarre irregular nuclei and permanent nucleoli, entrapped in fibrotic stroma, consistent with carcinoma, H&E stain, 400x. (IV) Metastatic carcinoma cells are highlighted by immune-stain (right lower corner); residual uninvolved lymph node shows no immunopositivity (left upper corner), wide spectrum cytokeratin immunostain, 100x.

74-year-old otherwise healthy male presented with a 3 × 4 cm^2^ painful nodule on the upper back. The lesion was initially diagnosed as a lipoma and surgically removed. However, six months after resection, the patient developed thoracic back pain. Thoracic MRI revealed an infiltrative mass of T3 to T5 vertebrae. A WLE with T3 to T5 laminectomy was performed, and the excision specimen was interpreted as squamous cell carcinoma. One year later, his back pain reemerged, and the patient developed bilateral lower extremity weakness, prompted a repeat MRI, which demonstrated 4.7 × 5.4 × 7.5 cm^3^ soft tissue cystic mass at the T3 to T5 vertebral level invading the neural foramina bilaterally as well as an enlarged right axillary lymph node (Figure 1(a)). Fine needle aspiration of the lymph node showed a high-grade carcinoma showing papillary and tubular architectural features. The tumor cells were immunoreactive for high molecular weight keratin (CK5/6), wide spectrum cytokeratin, and p63 immuno-stains (Figure 1(b)). Taken together, the morphologic and immunophenotypic features supported the diagnosis of SCACP.

**Figure 2:**
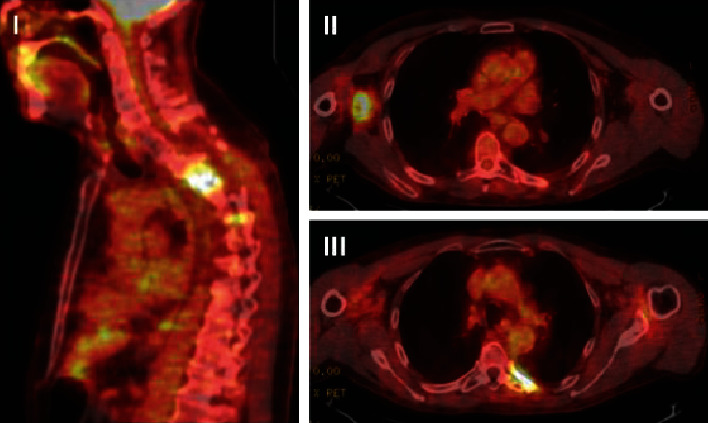
Positron emission tomography (PET) scan images 12 months after chemoradiotherapy. Hypermetabolic activity in the left paraspinal region at the level of T4-T5 (I), in the right axilla (II), and in the left sixth rib medially (III).

The patient was deemed not a surgical candidate and was treated with three cycles of carboplatin and paclitaxel with concurrent intensity-modulated radiation therapy (IMRT), which resulted in disease stabilization and significant improvement of his neurological symptoms. However, he developed right arm pain and weakness within a year of treatment, and the repeat imaging showed an increase in size of the right axillary lymph node and stable disease in his spine (Figure 2). The lymph node was resected and sent for the next generation sequencing (The FoundationOne assay interrogates 315 genes as well as introns of 28 genes involved in rearrangements), which revealed a total of 18 genomic alterations associated with potential benefit from targeted therapeutics (Table 1). PD-L1 expression was identified in 70% of tumor cells using Tumor Proportion Score (TPS). Patient was offered checkpoint inhibitor-based therapy that he declined.

## 3. Discussion

SCACP is an extremely rare cutaneous neoplasm of the apocrine or eccrine sweat glands. Due to the rarity of this entity, clinical and pathologic diagnosis is challenging. However, solid and cystic glandular structures with cribriform and tubular architecture along with CK5/6, pankeratin, and p63 immuno-profile set apart SCACP from other cutaneous malignancies.

WLE has been the mainstay treatment for localized SCACP; however, high recurrence rate (30-40%) following surgery has been reported [4, 6]. For unresectable or metastatic disease, no standard treatment has yet been established. To date, there has been only one case reported, in which a cisplatin chemoradiation was delivered, and the patient was rendered disease free for 11 months [7]. Our patient achieved disease control for 12 months with carboplatin and paclitaxel concurrently with IMRT.

To determine potential therapeutic strategies, the molecular profile of the tumor was requested, which revealed 18 genomic alterations associated with potential clinical benefit (Table 1). Among these alterations, ERBB4 R293W has been previously observed in several cancers including malignant melanoma, which was found to be sensitive to the ERBB inhibitor, lapatinib *in vitro* [8]. Another genomic alteration which may indicate biological and therapeutic relevance is PIK3CA E453K [9]. PIK3CA activating mutations or amplification may predict sensitivity to inhibitors of PI3K or its downstream signaling pathway (the PI3K/Akt/mTOR pathway) [10]. Moreover, the impaired function of CDKN2A results in cell cycle dysregulation [11] and clinical benefit from CDK4/6 inhibitors has been shown in patients harboring CDKN2A alterations [12, 13]. Although these are potentially interesting findings, further studies will need to be performed to validate these genomic alterations.

Lastly, the efficacy of immunotherapy in the treatment of SCACP has not yet been demonstrated. To our knowledge, this is the first report of PD-L1 expression in SCACP, and the result suggests the possibility of using checkpoint inhibitors in this otherwise difficult to treat disease.

## 4. Conclusion

We report a case of metastatic SCACP, its histopathological and genomic characterization, and response to the combined modality of treatment. The opportunity of targeted therapeutics and immunotherapy exist as for metastatic SCACP. Reporting molecular profile of the rare tumors with no established standard treatment options should be encouraged.

## Figures and Tables

**Table 1:** Molecular characterization of the patient's tumor.

Gene	Gene function	Mutation	Mutation description	Targeted therapy
ARID2	Activation of gene expression by nuclear receptors	Q725^∗^	Nonsense	None
CDKN2A	Tumor suppressor	p16INK4a Q50^∗^	Nonsense	Palbociclib
ERBB4	Cell proliferation and apoptosis	R393W	Missense	Afatinib, Erlotinib, Lapatinib
FAT1	Inhibition of transcriptional activity	G758fs	Frameshift	None
FGF10	Regulation of cell growth, tumor growth and invasion	Amplification	—	None
FGFR1	Regulation of the cell cycle and angiogenesis	Q775^∗^	Nonsense	Ponatinib, Pazopanib
GNA11	Modulation of transmembrane signaling system	R183C	Missense	Trametinib
KDM5C	Control gene expression	Q919^∗^	Nonsense	None
MAGI2	Inhibition of cell migration and proliferation	Q117^∗^	Nonsense	None
NFKBIA	Tumor suppressor	Amplification	—	None
NOTCH1	Regulation of cell fate, proliferation and apoptosis	6181-1G >A	Intronic	None
PIK3CA	Regulation of cell growth, proliferation, and survival	E453K	Missense	Everolimus, Temsirolimus
RAC1	Regulation of tumor angiogenesis, metastasis and cell growth	P29S^∗^	Nonsense	None
RICTOR	Encoding an mTOR-binding protein	Amplification	—	Omipalisib
SLIT2	Tumor suppressor	D1445H	Missense	None
TERT	Regulation of chromosomal length	124C >T	Intronic	None
TP53	Tumor suppressor	Q331^∗^	Nonsense	Adavosertib
TP53	Tumor suppressor	Q375^∗^	Nonsense	Adavosertib
